# An Interface ASIC Design of MEMS Gyroscope with Analog Closed Loop Driving

**DOI:** 10.3390/s23052615

**Published:** 2023-02-27

**Authors:** Huan Zhang, Weiping Chen, Liang Yin, Qiang Fu

**Affiliations:** 1 MEMS Center, Harbin Institute of Technology, Harbin 150001, China; 2 Key Laboratory of Micro-Structures Manufacturing, Harbin Institute of Technology, Ministry of Education, Harbin 150001, China

**Keywords:** MEMS gyroscope, electrical model, on-chip temperature sensor, digital output, circuit design

## Abstract

This paper introduces a digital interface application-specific integrated circuit (ASIC) for a micro-electromechanical systems (MEMS) vibratory gyroscope. The driving circuit of the interface ASIC uses an automatic gain circuit (AGC) module instead of a phase-locked loop to realize a self-excited vibration, which gives the gyroscope system good robustness. In order to realize the co-simulation of the mechanically sensitive structure and interface circuit of the gyroscope, the equivalent electrical model analysis and modeling of the mechanically sensitive structure of the gyro are carried out by Verilog-A. According to the design scheme of the MEMS gyroscope interface circuit, a system-level simulation model including mechanically sensitive structure and measurement and control circuit is established by SIMULINK. A digital-to-analog converter (ADC) is designed for the digital processing and temperature compensation of the angular velocity in the MEMS gyroscope digital circuit system. Using the positive and negative diode temperature characteristics, the function of the on-chip temperature sensor is realized, and the temperature compensation and zero bias correction are carried out simultaneously. The MEMS interface ASIC is designed using a standard 0.18 μM CMOS BCD process. The experimental results show that the signal-to-noise ratio (SNR) of sigma-delta (ΣΔ) ADC is 111.56 dB. The nonlinearity of the MEMS gyroscope system is 0.03% over the full-scale range.

## 1. Introduction

Micromechanical gyroscope is a kind of inertial sensor which uses Coriolis force effect to detect angular velocity, angle and angular acceleration of external input. It can detect changes in the external state of the sensor without external reference information [1,2]. In addition, micromechanical gyroscopes and accelerometers can be combined to form micro-inertial measurement units (MIMU) with a small volume, light weight and high reliability, thus becoming high-performance navigation microsystems [3,4]. The traditional rotor-type mechanical gyroscope has been unable to meet the requirements of high-performance weapons and industrial applications, due to its large volume, high power consumption, low bandwidth and high cost. Although fiber optic gyroscope and laser gyroscope have the advantages of high precision and high performance, their large volume, high power consumption and high cost also limit their application range [5,6]. Therefore, the MEMS capacitor sensor, as an important sensor system, is widely used in aerospace, industrial control, military and medical fields [7,8,9]. In recent years, with the huge demand in the consumer electronics market, high performance gyro systems such as frame type [10], disk type [11,12,13] and butterfly type [14,15,16] have been researched and developed successively. Therefore, how to further improve the overall performance of the MEMS gyroscope and develop its potential performance has become a research hotspot in recent years.

In order to improve the stability of the gyroscope drive circuit and the performance of the whole machine, many studies have developed different circuit schemes of the gyroscope interface circuit. At present, the drive circuit mainly includes an analog open-loop drive circuit and digital non-self-excited closed-loop drive [17,18,19]. However, due to the poor stability of the drive circuit in the first method, the stable oscillation of the central proof mass cannot be realized with the change in external temperature [20,21]. Although the second method can guarantee the driving stability, it cannot be applied to the gyro’s sensitive structure with different resonant frequencies. However, most of the previous studies have some imperfections, and the interface circuit developed is of low integration, mostly in the form of a hybrid package of FPGA and PCB [22,23,24]. The simulation closed-loop self-excited drive method can realize the gyroscope stable self-excited oscillation and improve the driving stability and temperature adaptability of the MEMS gyroscope [25,26]. In addition, it is very necessary to use the on-chip temperature sensor to realize the zero bias and temperature drift compensation function of the calibration gyro, so as to further improve the integration of the gyro interface circuit. Therefore, it is a valuable method to use highly integrated interface ASIC to realize the measurement and control of the gyroscope [27,28].

This paper introduces a digital interface ASIC for a MEMS vibrating gyroscope. By using a 0.18 μM CMOS BCD process, the driving circuit and sense circuit are integrated into an ASIC, which realizes the miniaturization and low power consumption of the MEMS gyro system. Firstly, in order to realize the design and optimization of the whole gyro system, the equivalent electrical model of the MEMS gyro’s sensitive structure is modeled and analyzed. The co-simulation analysis of the sensitive structure and interface circuit of the gyro is carried out by SIMULINK, and the feasibility of the circuit design is verified. Then, the design principle of the key circuit module of the MEMS gyro interface circuit is analyzed, and the performance index of the key circuit module is tested and simulated. The designed ΣΔ ADC realizes the digital output of the angular velocity, and the maximum SNR of the ΣΔ modulator is 115.6 dB. In addition, based on the diode temperature characteristics, the on-chip temperature sensor is constructed, and the on-chip temperature compensation and zero bias correction are carried out for the gyroscope. Finally, the performance of the gyroscope is tested experimentally. The test results show that the interface ASIC can realize the high precision measurement of the gyro angular velocity.

The content of this work is as follows: Section 2 introduces the working principle of the MEMS gyroscope, and establishes the electrical equivalent simulation model of the gyro’s sensitive structure. Section 3 introduces the design and simulation of the system-level model of the interface ASIC of the MEMS gyroscope. Section 4 introduces the design principle of interface ASIC key circuit module in detail. The experimental results of the MEMS gyroscope system are analyzed in Section 5. A comprehensive conclusion is given in Section 6.

## 2. Working Principle of the MEMS Gyroscope and Electrical Model Establishment

The MEMS gyroscope works on the principle of Coriolis force mechanics. When the angular velocity signal is input, the proof mass will produce forced vibration in the tangential direction of resonance. The change in the sense direction capacitance of the gyroscope can realize the detection of the input angular velocity. In order to realize the co-simulation of the gyro’s sensitive structure and transistor-level interface circuit, it is necessary to establish the electrical equivalent model of the sensitive structure.

### 2.1. Dynamics Principle of the MEMS Gyroscope

**Figure 1:**
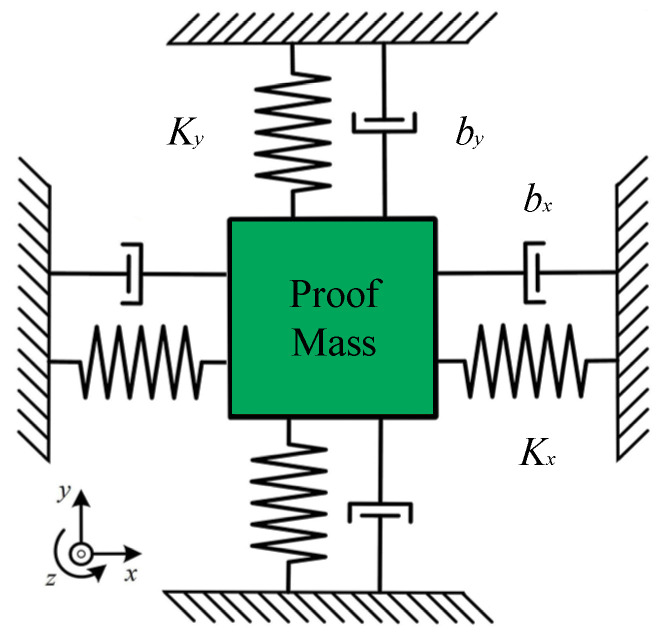
Mechanical model of MEMS gyroscope.

The sensitive structure of the MEMS gyroscope is to realize angular velocity detection by using Coriolis force mechanics principle. Figure 1 below shows the mechanical model of the working principle of the gyroscope. *k_x_* and *k_y_* represent the elastic coefficient in the *x* and *y*-axis direction, respectively, while *b_x_* and *b_y_* represent the damping coefficient of sensitive structure, respectively. The electrostatic force *F_d_* as gyroscope natural frequency ω_x_ is applied in the *x*-axis direction to driving the proof mass to do simple harmonic vibration in the *x*-axis. When there is an angular velocity input in the *z*-axis direction, a gyro system rotating about the *z*-axis creates the Coriolis force in the non-inertial system.

For the gyroscope driving mode, the sum of the external forces on the mass in the *x*-axis direction is equal to the product of acceleration and mass. According to Equation (1), the acceleration of the proof mass in the driving direction of the gyro system can be obtained, and the external force of the proof mass in the *x*-axis direction can be obtained, including the driving force, spring elastic force and damping force.

(1) $$F_{x}=F_{d}-k_{x}x-b_{x}\dot{x}$$

When angular velocity Ω is input along the *z*-axis, the motion equation of proof mass along the *x*-axis direction under the action of inertia force coupling is:

(2) $$F_{d}-k_{x}x-b_{x}\dot{x}=m\ddot{x}-m\Omega^{2}x-2m\Omega \dot{y}-m\dot{\Omega }y$$

Because the resonant displacement of the gyroscope mass is very small in the *x*-axis direction, $m\dot{\Omega }y$ can be ignored. The Coriolis force $2m\dot{\Omega }y$ at the driving end caused by the resonance of the detecting end is far less than the driving force *F_d_*, which can also be ignored. Ignoring *m*Ω^2^*x*, the motion equation in the driving direction of the gyroscope can be further obtained as:

(3) $$m\ddot{x}+k_{x}x+b_{x}\dot{x}=F_{d}$$

The motion equation of the proof mass in the *y*-axis direction is:

(4) $$-k_{y}y-b_{y}\dot{y}=m\ddot{y}-m\Omega^{2}y+2m\Omega \dot{x}+m\dot{\Omega }x$$

Unlike the driving direction, there is no external driving force in the sense direction. The Coriolis force generated by the driving resonance cannot be ignored, and the equation of motion in the sense direction of the gyroscope can be written as Equation (5):

(5) $$m\ddot{y}+k_{y}y+b_{y}\dot{y}=-2m\Omega \dot{x}$$

### 2.2. Operating Principle of the MEMS Gyroscope Driving Mode

**Figure 2:**
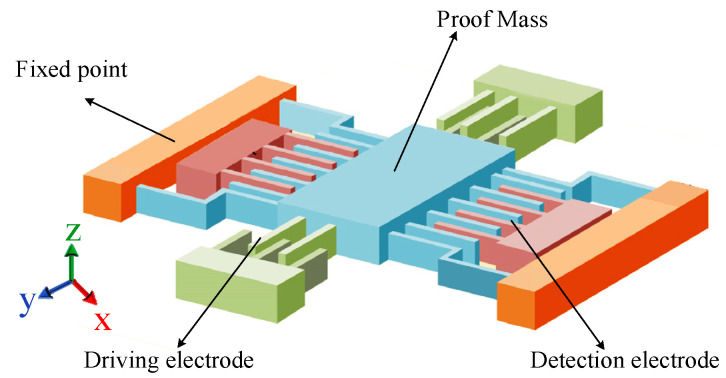
Schematic diagram of mechanically sensitive structure of MEMS gyroscope.

Figure 2 shows the structure diagram of the single proof mass gyroscope. *xoy* is the in-plane direction, and the proof mass can perform simple harmonic vibration along the driving *x*-axis direction when driven by electrostatic force [29,30]. The sense detection is the *y*-axis direction, and the *z*-axis direction is the angular velocity direction to be measured.

When the AC frequency of the voltage applied to the driving electrode is the same as the natural frequency of the mass in the *x*-axis direction, the central proof mass of the gyro starts to generate harmonic motion. The dynamic equation of displacement in the driving direction can be written by Equation (6).

(6) $$M_{d}\frac{d^{2}x}{dt^{2}}+\lambda_{d}\frac{dx}{dt}+K_{d}x=F_{total}$$

where the equivalent mass in the *x*-axis direction is *M_d_*, the damping force coefficient and the elastic coefficient in the *x*-axis direction are *λ_d_* and *K_d_*, respectively, and *F_tot_* represents the force in the *x*-axis direction.

If the resonant angular frequency of the gyroscope is $w_{d}=\sqrt{\frac{K_{d}}{M_{d}}}$, and damping ratio is $\xi_{d}=\frac{\lambda_{d}}{2M_{d}w_{d}}$. The quality factor of the gyroscope is $Q_{d}=\frac{1}{2\xi_{d}}=\frac{M_{d}w_{d}}{\lambda_{d}}$, then Equation (6) can be written as:

(7) $$\frac{d^{2}x}{dt^{2}}+2\xi_{d}w_{d}\frac{dx}{dt}+w_{d}{}^{2}x=\frac{F_{total}\sin wt}{M_{d}}$$

It can be obtained by solving Equation (8), and *B_d_* and *φ_d_* are Equations (9) and (10), respectively:

(8) $$\begin{array}{l} x(t)=\frac{F_{total}/M_{d}}{\sqrt{{\left(w_{d}{}^{2}-w^{2}\right)}^{2}+4\xi_{d}{}^{2}w_{d}{}^{2}w^{2}}}\left[(w_{d}{}^{2}-w^{2})\sin wt-2\xi_{d}w_{d}w\cos wt\right]\\ =\frac{F_{total}\sin(wt+\varphi_{d})/M_{d}}{w_{d}{}^{2}\sqrt{{\left(1-\frac{w^{2}}{w_{d}{}^{2}}\right)}^{2}+4\xi_{d}{}^{2}\frac{w^{2}}{w_{d}{}^{2}}}}=B_{d}\sin(wt+\varphi_{d}) \end{array}$$

(9) $$B_{d}=\frac{F_{total}}{M_{d}w_{d}{}^{2}\sqrt{{\left(1-\frac{w^{2}}{w_{d}{}^{2}}\right)}^{2}+{\left(\frac{w}{Q_{d}w_{d}}\right)}^{2}}}$$

(10) $$\varphi_{d}=arctg\frac{-2\xi_{d}w_{d}w}{w_{d}{}^{2}-w^{2}}$$

When the frequency of the differential voltage applied is equal to the natural frequency of the mass, the proof mass of the gyroscope moves in simple harmonic motion under the action of the driving voltage. The gyroscope driving circuit adopts the closed-loop self-excited driving scheme, which can obtain the resonant motion with the natural frequency of the proof mass and ensure the stability of the driving circuit to meet the requirements.

### 2.3. Working Principle of the MEMS Gyroscope Sense Mode

When the angular velocity is input, the Coriolis force on the proof mass in the *y*-axis direction is:

(11) $$F_{c}=2M\Omega B_{d}w\cos(wt+\varphi_{d})$$

Therefore, the displacement dynamics equation of the gyroscope sense direction can be expressed by Equation (12).

(12) $$M_{s}\frac{d^{2}y}{dt^{2}}+\lambda_{s}\frac{dy}{dt}+k_{s}y=2B_{d}M_{s}\Omega w\cos(wt+\varphi_{d})$$

where the equivalent mass of the sense direction is *M_s_*, and the damping coefficient and elastic coefficient of the *y*-axis are *λ_s_* and *K_s_*, respectively.

If the gyroscope’s sense direction of oscillation angular frequency is $\omega_{s}=\sqrt{\frac{K_{s}}{M_{s}}}$, and damping ratio is $\xi_{s}=\frac{\lambda_{s}}{2M_{s}w_{s}}$.

(13) $$y(t)=B_{t}(t)+B_{s1}\cos[(w-w_{i})t+\varphi_{s1}+\varphi_{d}]+B_{s2}\cos[(w+w_{i})t+\varphi_{s2}+\varphi_{d}]$$

*B_s_*_1_, *φ_s_*_1_, *B_s_*_2_ and *φ_s_*_2_ can be obtained by the following expressions, respectively.

(14) $$\begin{array}{l} B_{s1}=\frac{B_{d}\Omega_{0}\omega }{\omega_{s}{}^{2}\sqrt{{\left(1-\frac{{\left(\omega -\omega_{i}\right)}^{2}}{\omega_{s}{}^{2}}\right)}^{2}+4\xi_{s}{}^{2}{\left(\frac{\omega -\omega_{i}}{\omega_{s}}\right)}^{2}}}\\ B_{s2}=\frac{B_{d}\Omega_{0}\omega }{\omega_{s}{}^{2}\sqrt{{\left(1-\frac{{\left(\omega +\omega_{i}\right)}^{2}}{\omega_{s}{}^{2}}\right)}^{2}+4\xi_{s}{}^{2}{\left(\frac{\omega +\omega_{i}}{\omega_{s}}\right)}^{2}}}\\ \varphi_{s1}=arctg\frac{2\xi_{s}\omega_{s}\left(\omega -\omega_{i}\right)}{\omega_{s}{}^{2}-{\left(\omega -\omega_{i}\right)}^{2}}\\ \varphi_{s2}=arctg\frac{2\xi_{s}\omega_{s}\left(\omega +\omega_{i}\right)}{\omega_{s}{}^{2}-{\left(\omega +\omega_{i}\right)}^{2}} \end{array}$$

The first term on the right of Equation (13) is a transient term and can be ignored, and the second and third terms are carrier amplitude modulation waves. Equation (13) can be simplified as:

(15) $$\begin{array}{l} y\left(t\right)=B_{s1}\cos\left[\left(\omega -\omega_{i}\right)t+\varphi_{s1}+\varphi_{d}\right]+B_{s2}\cos\left[\left(\omega +\omega_{i}\right)t+\varphi_{s2}+\varphi_{d}\right]\\ =\left(B_{s1}+B_{s2}\right)\cos\left(\omega_{i}t+\frac{\varphi_{s2}-\varphi_{s1}}{2}\right)\cos\left(\omega t+\frac{\varphi_{s2}+\varphi_{s1}}{2}+\varphi_{d}\right)\\ +\left(B_{s1}-B_{s2}\right)\sin\left(\omega_{i}t+\frac{\varphi_{s2}-\varphi_{s1}}{2}\right)\sin\left(\omega t+\frac{\varphi_{s2}+\varphi_{s1}}{2}+\varphi_{d}\right) \end{array}$$

$\cos(\omega t+\frac{\varphi_{s1}+\varphi_{s2}}{2}+\varphi_{d})$ can be used as the demodulation signal of the angular velocity sensitive detection signal in Equation (15). After the double frequency signal is filtered by the low-pass filter, the final output of the gyroscope can be written:

(16) $$y\left(t\right)=\frac{1}{2}\left(B_{s1}+B_{s2}\right)\cos\left(\omega_{i}t+\frac{\varphi_{s2}-\varphi_{s1}}{2}\right)$$

The gyroscope output signal and input angular velocity frequency is the same, and the amplitude is proportional. The signal can reflect the input angular velocity information, and it can reflect the external angular velocity characteristics [31].

### 2.4. Equivalent Electrical Model of the MEMS Gyroscope

The working principle of the gyroscope can be understood through gyroscope dynamic equation, but the mechatronics simulation design of the gyroscope cannot be completed. In order to ensure that the MEMS gyroscope integrated ASIC can match the actual mechanically sensitive structure of the gyroscope, it is necessary to realize the joint simulation of the gyroscope’s mechanically sensitive structure and interface circuit in the circuit design and simulation stage. Therefore, it is necessary to establish the equivalent electrical model of the MEMS gyroscope’s mechanically sensitive structure. The MEMS gyroscope is equivalent to the spring harmonic oscillator model. The spring oscillator is subject to electrostatic driving force and Coriolis force, respectively, in driving and sense directions. The dynamic equation of the gyroscope can be expressed by Equation (17).

(17) $$\left\{\begin{array}{l} m_{d}\ddot{x}+b_{d}\dot{x}+k_{d}x=F_{d}\\ m_{s}\ddot{y}+b_{s}\dot{y}+k_{s}y=F_{c} \end{array}\right.$$

By taking the derivative of Equation (17), Equation (18) can be further calculated.

(18) $$\left\{\begin{array}{l} m_{d}\frac{d^{2}v_{x}}{dt^{2}}+b_{d}\frac{dv_{x}}{dt}+k_{d}v_{x}=\frac{dF_{d}}{dt}\\ m_{s}\frac{d^{2}v_{y}}{dt^{2}}+b_{s}\frac{dv_{y}}{dt}+k_{s}v_{y}=\frac{dF_{c}}{dt} \end{array}\right.$$

**Figure 3:**
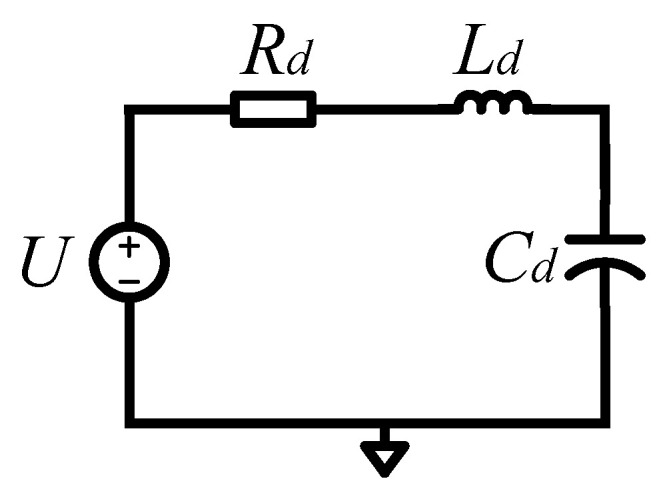
Equivalent electrical model of MEMS gyroscope.

Equation (18) contains the second-order differential term, first-order differential term and general solution term. An electrical model consists of the resistor *R*, the capacitor *C*, the inductor *L* and a constant voltage source *U* in series, as shown in Figure 3. According to Kirchhoff’s voltage law, its voltage–current relationship equation can be written as Equation (19).

(19) $$L\cdot \frac{di}{dt}+\frac{1}{c}\cdot \int_{0}^{t}i\cdot dt+R\cdot i=U$$

It takes the derivative of both sides of Equation (20).

(20) $$L\cdot \frac{d^{2}i}{dt^{2}}+R\cdot \frac{di}{dt}+\frac{1}{c}\cdot i=\frac{dU}{dt}$$

It can be seen from Equation (20) that the voltage–current equation of an electrical model of LCR formed in series also contains second-order differential terms, first-order differential terms and general solution terms. The electrical parameters of such a series LCR electrical model can correspond one to one with the physical parameters in the mechanical dynamics equation of a gyroscope, which has the same characteristics in essence.

**Figure 4:**
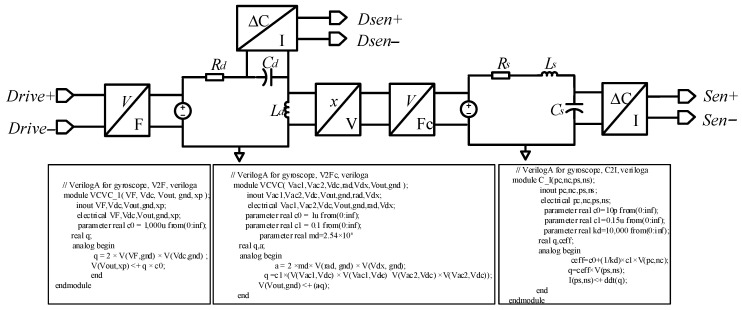
Equivalent electrical model of mechanically sensitive structure of gyroscope.

The simultaneous Equations (18) and (20) can realize the transformation of the MEMS gyroscope’s mechanically sensitive structural mechanics model from mechanical domain to electrical domain, and the corresponding relationship is written in Table 1. According to the equivalent electrical model of the driving and sense direction of the MEMS gyroscope and the corresponding relationship between its physical–electrical parameters, the equivalent electrical model of the mechanically sensitive structure of the MEMS gyroscope can be obtained, as shown in Figure 4.

The equivalent electrical model of the MEMS gyroscope’s sensitive structure is realized by the combination of resistance and tolerance components and Verilog-A module. The electrostatic driving force (*F_d_*) of the driving loop makes the MEMS gyroscope’s central proof mass oscillate in stable amplitude along the driving direction. The amount of charge on the capacitor (*Q_d_*) represents the displacement in the driving direction, which is converted into velocity and multiplied by the Coriolis force coefficient to obtain the Coriolis force (*F_c_*) in the sense direction. The Coriolis force causes the gyroscope’s sensitive structure to produce a certain displacement of sense direction, and the amount of charge on *C_s_* reflects the displacement, so that the external input angular velocity (Ω) can be calculated.

## 3. System-Level Modeling Design and Simulation

**Figure 5:**
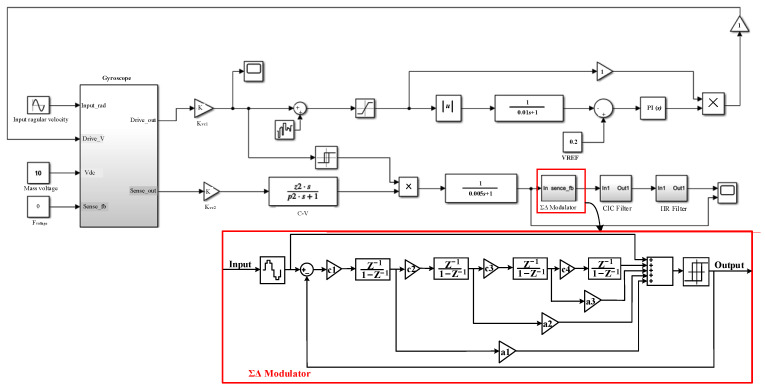
Schematic diagram of gyroscope system-level simulation model.

The MEMS gyroscope is a micro-electromechanical system, which includes a mechanically sensitive structure and interface circuit. Based on Simulink simulation platform, the co-simulation of the mechanical system and electrical system can be realized by using the sensitive structure model. The gyroscope system-level circuit can verify the interface circuit design scheme and optimize the key circuit module design parameters. Figure 5 shows the schematic diagram of the MEMS gyroscope simulation model, which mainly includes the MEMS gyroscope’s mechanically sensitive structure module, driving circuit and sense circuit module. The system-level model is the premise of transistor level interface circuit design, which can verify the accuracy of system circuit design. This section mainly analyzes the driving circuit and sense circuit design principle and simulation results analysis.

### 3.1. Modeling and Simulation of Driving Circuit

**Figure 6:**
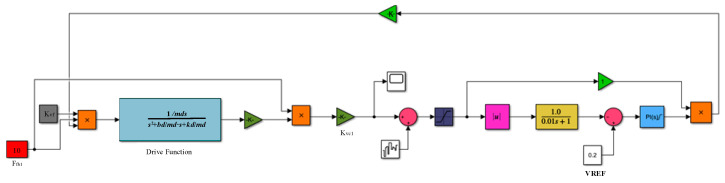
System-level model of gyroscope driving circuit.

When a voltage of the same frequency as the natural frequency of the gyroscope driving mode is applied to the driving electrode, the resulting electrostatic driving force drives the MEMS gyroscope to complete self-excited oscillation. The driving circuit adopts the analog circuit closed-loop scheme, and the driving circuit makes the gyroscope oscillate steadily along the driving direction. Because of the high-quality factor of the gyroscope, it has good frequency selection characteristics. By applying the driving detection signal to the driving electrode through a positive feedback loop, the gyroscope is self-excited, and the driving displacement increases continuously. If the driving displacement is too large, it will even destroy the sensitive structure of the gyroscope drive and cause permanent damage to the mechanical structure. Therefore, it is necessary to introduce negative feedback into the driving loop. When the driving displacement amplitude is too large, the loop gain can be automatically adjusted to reduce the driving feedback voltage and thus the driving displacement. AGC can ensure that the driving loop vibrates according to the reference voltage amplitude, and the PI controller can ensure that the simulation closed-loop has good stability. Figure 6 shows the system-level model of the MEMS gyroscope driving circuit.

**Figure 7:**
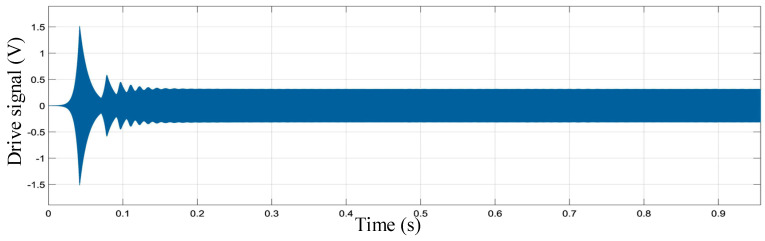
Simulation diagram of self-excited oscillation of the driving circuit.

First, the charge amplifier converts the capacitance change generated by the gyroscope’s sensitive structure into a voltage signal. Then, the voltage signal is extracted from the amplitude of the voltage signal by the rectifier and the low-pass filter. The amplitude of the voltage signal is controlled by the PI controller to ensure that the voltage in the driving loop vibrates according to the set amplitude. Finally, the driving voltage signal multiplied by the amplitude is fed back to the mechanically sensitive structure to complete the closed-loop design of the driving loop. As shown in Figure 7, the simulation results of the driving circuit model show that the voltage signal of the driving loop can complete the self-excited oscillation with steady amplitude at 0.2 s. By adjusting the PI controller parameters, the gain and phase margin of the driving loop can be dynamically adjusted. The simulation results show that the overshoot of the PI controller is appropriate in the process of self-excited startup. The drive circuit can complete the fast and stable self-excited driving.

### 3.2. Modeling and Simulation of Sense Circuit

**Figure 8:**
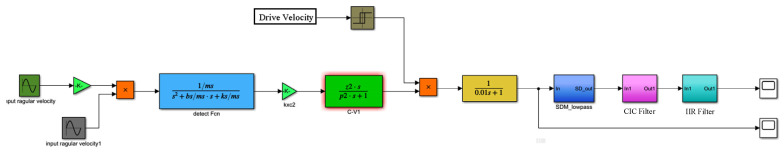
System-level model of gyroscope sense circuit.

The sense circuit adopts switch demodulation to reduce the consumption of circuit hardware resources. The sensing circuit uses single bit quantized ΣΔ modulator to make the digital output angular velocity signal have high linearity. The digital output side provides back-end circuit for signal correction and compensation processing, which can make the sensor compatible with different communication interface protocols. The third-order CIC filter and the second-order IIR comprise a fifth-order cascade filter, and the bitstream output of the ΣΔ modulator is converted into the 24-bit digital signal output by the digital filter. Figure 8 is a system-level model of the gyroscope sense circuit.

**Figure 9:**
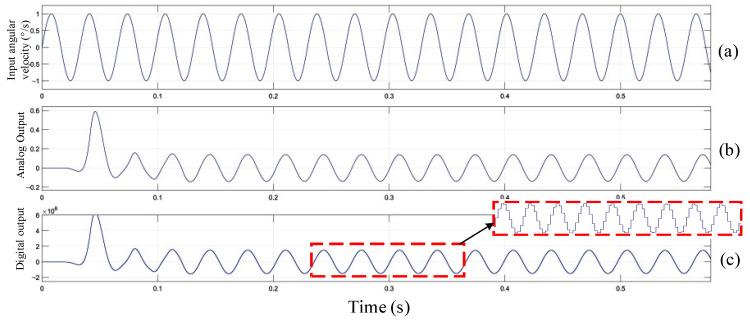
Sense circuit input and output simulation diagram: (**a**) Input angular velocity; (**b**) Analog output; (**c**) Digital output.

In order to verify the correctness of the sense circuit design, it is necessary to simulate and analyze the system-level model of the MEMS gyroscope sense circuit. When the input angular velocity signal is 1°/s and the frequency is 10 Hz, the input angular velocity signal and analog and digital output signal are as shown in Figure 9b,c, respectively. The simulation waveform verifies that the output angular velocity of the induction loop is correct, and the feasibility of the MEMS gyroscope sense circuit is further verified.

## 4. Design of the MEMS Gyroscope Interface Circuit

**Figure 10:**
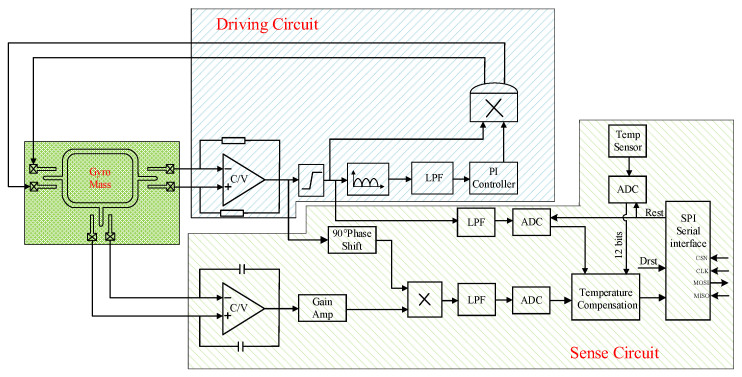
Topology structure of MEMS gyroscope interface circuit.

The integrated design of the MEMS gyroscope’s mechanically sensitive structure and interface circuit can ensure the sensor system has the advantages of miniaturization and low power consumption. Therefore, the driving loop of the gyroscope interface circuit uses a self-excited closed-loop analog circuit, and the sense circuit uses a digital output scheme. Figure 10 shows the topology structure of the MEMS gyroscope interface circuit. When the driving loop performs amplitude stabilization resonance, the Coriolis force causes quantitative displacement motion along the sense direction. The resulting displacement will cause a change in the capacitance charge of the sense electrode, which is detected by the charge amplifier. The sense signal is output to ΣΔ ADC after switched-phase sensitive demodulation and low-pass filtering, and ADC converts the analog signal to digital signal output. In order to improve the gyroscope temperature drift and zero bias stability, the on-chip temperature sensor is used to compensate the temperature and zero bias of the system.

### 4.1. Design of AGC Circuit Module

**Figure 11:**
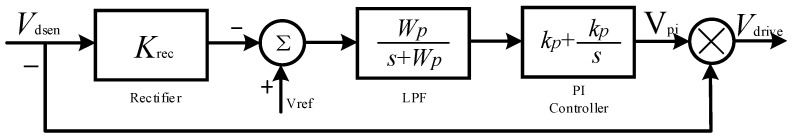
Schematic diagram of the AGC module of the driving circuit.

The PI controller is the most important module of the AGC circuit. The PI controller has a fast convergence speed and can eliminate the static error of the automatic control. In addition, the PI controller is not sensitive to high frequency noise, and it meets the performance design requirements of the driving loop. Figure 11 shows the circuit schematic diagram of the AGC module. The AGC circuit consists of three modules: peak detection, PI controller and nonlinear multiplier. The peak detection circuit module is composed of a full wave rectifier circuit and a low-pass filter, which is used to detect the driving loop amplitude. This amplitude information is the displacement amplitude of self-excited oscillation in the driving direction. The PI controller compares the amplitude of *V_dsen_* with the static error of the reference voltage *V_ref_*, which is amplified into the gain of the output voltage *V_PI_* which is used to control the nonlinear multiplier. *V_dse_*_n_ adjusts the gyroscope driving displacement by multiplying a voltage gain by a nonlinear multiplier and feeding the drive feedback voltage *V_drive_* to the driving feedback electrode. The PI controller can eliminate the static error between the amplitude of *V_dsen_* and the reference voltage *V_ref_* through negative feedback, in order to ensure that the driving displacement is controlled at the reference voltage.

**Figure 12:**
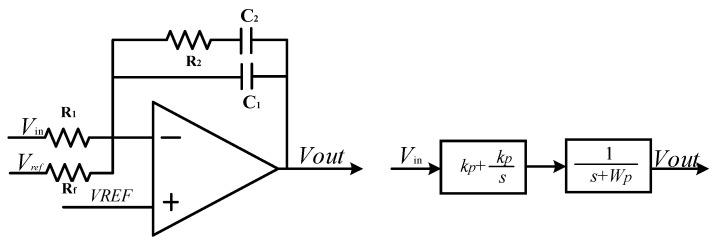
Circuit schematic diagram of the PI controller.

The PI controller compares the driving displacement amplitude obtained after low-pass filtering with the reference voltage, and outputs the voltage signal to the voltage-controlled nonlinear multiplier gain. Adjust the driving displacement amplitude to realize the voltage-controlled gain function of the AGC module. Figure 12 shows the highly integrated PI controller designed. According to the PI controller circuit structure, its transfer function can be obtained by Equation (21).

(21) $$\frac{V_{out}}{V_{in}}=-\left[\frac{R_{2}}{R_{1}}\frac{C_{2}}{C_{1}+C_{2}}+\frac{1}{R_{1}\left(C_{1}+C_{2}\right)}\right]\cdot \frac{1}{1+\frac{sR_{2}C_{1}C_{2}}{C_{1}+C_{2}}}$$

*k_p_* is the proportion term and is the integral term, and *w_p_* is the pole of the low-pass filter.

(22) $$\left\{\begin{array}{l} k_{P}=\frac{R_{2}}{R_{1}}\cdot \frac{C_{2}}{C_{1}+C_{2}}\\ k_{I}=\frac{1}{R_{1}\left(C_{1}+C_{2}\right)}\\ \omega_{p}=\frac{C_{1}+C_{2}}{R_{2}C_{1}C_{2}} \end{array}\right.$$

The PI controller’s proportional and integral terms are added in the form of adder, and the input and output ends of the operation amplifier are paralleled capacitor feedback to achieve a first-order low-pass filter. A PI controller circuit with a single operational amplifier can be realized, greatly saving power consumption and layout area.

### 4.2. Design and Analysis of Nonlinear Multiplier

**Figure 13:**
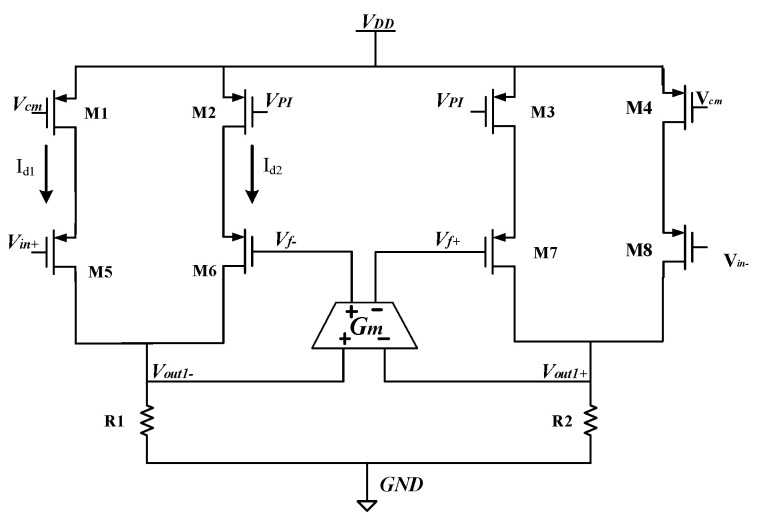
The transistor-level circuit structure diagram of nonlinear multiplier.

Nonlinear multiplier is one of the key modules of the MEMS gyroscope driving circuit. It implements the function of driving loop gain control in AGC circuit. Figure 13 shows the transistor-level circuit of the nonlinear multiplier. *V_in+_* and *V_in−_* are the driving detection signal and anti-trust signal. *V_PI_* is the output voltage signal of the highly integrated PI controller, which represents the static error of driving displacement amplitude and reference voltage. *V_cm_* is the common mode level of the driving circuit and determines the static characteristics of the nonlinear multiplier circuit. *V_drive+_* and *V_drive−_* are the driving feedback voltage signal by the nonlinear multiplier. The nonlinear multiplier adopts full symmetry structure and can effectively suppress harmonic distortion. Only half of the circuit can be analyzed when analyzing the principle of the circuit.

First, *V_cm_* is set so that transistor *M*_1_ works in the linear region. Transistors *M*_5_ and *M*_1_ and resistor *R*_1_ form a common source amplifier with negative feedback from the P-type transistor input. The equivalent resistance of the linear region of transistor *M*_1_ can be calculated as:

(23) $$R_{M1}=\frac{1}{\mu_{p}C_{ox}{\left(\frac{W}{L}\right)}_{1}\left(V_{DD}-V_{CM}-\left|V_{TH}\right|\right)}$$

*G_M_*_5_ is the equivalent transconductance of transistor *M*_5_, and *G_M_*_5_ can be calculated from Equation (24).

(24) $$G_{M5}=\frac{g_{M5}}{1+g_{M5}R_{M1}}\approx \frac{1}{R_{M1}}=\mu_{p}C_{ox}{\left(\frac{W}{L}\right)}_{1}\left(V_{DD}-V_{CM}-\left|V_{TH}\right|\right)$$

Thus, the left half of the circuit current *I_d_*_1_ can be obtained.

(25) $$I_{d1}=G_{M5}V_{in+}=\mu_{p}C_{ox}{\left(\frac{W}{L}\right)}_{1}\left(V_{DD}-V_{CM}-\left|V_{TH}\right|\right)\cdot V_{in+}$$

When the gyroscope driving circuit is just powered on, the driving displacement of the gyroscope is very small. The displacement signal detected by the driving loop is very small, and the static error between the amplitude of the driving signal and the reference voltage *V_REF_* set by the PI controller is very large. Therefore, the PI controller output voltage *V_PI_* is full swing output, resulting in the transistor M_2_ in the cutoff region. In this case, the amplifier inside the nonlinear multiplier works in the open-loop state and has the characteristics of infinite open-loop gain. Since the input voltage of the nonlinear multiplier is the driving detection signal *V_dsen+_*, the input voltage of the operational amplifier can be calculated by Equation (26).

(26) $$V_{out1-}=I_{d1}R_{1}=G_{M1}V_{in+}R_{1}=\frac{R_{1}}{R_{M1}}V_{in+}=\frac{R_{1}}{R_{M1}}V_{dsen+}$$

When the open-loop gain is *A_OL_*, the driving feedback voltage can be obtained by Equation (27).

(27) $$V_{drive-}=A_{OL}V_{out1-}=A_{OL}\frac{R_{1}}{R_{M}}V_{dsen+}$$

The open-loop gain of the PI controller is infinite when the gyroscope is just powered on, and the output of operational amplifier is saturated. In this case, the driving feedback voltage drives the gyroscope’s sensitive structure with a full amplitude square wave signal of the same frequency. The gyroscopic driving mode can generate vibration quickly, and the driving displacement amplitude of the driving circuit increases rapidly.

When the amplitude of the driving displacement signal increases to a value close to the reference voltage *V_REF_*, the PI controller output voltage *V_PI_* decreases and is no longer the full-swing output. Therefore, when the transistor *M*_2_ is in the linear region, the transistor *M*_6_ tube, M_2_ and R_1_ will form a common source amplifier with negative source feedback.

(28) $$R_{M2}=\frac{1}{\mu_{p}C_{ox}{\left(\frac{W}{L}\right)}_{2}\left(V_{DD}-V_{CM}-\left|V_{TH}\right|\right)}$$

Equivalent transconductance *G_M_*_6_ of transistor M_6_ is expressed by Equation (29).

(29) $$G_{M6}=\frac{g_{M6}}{1+g_{M6}R_{M2}}\approx \frac{1}{R_{M2}}=\mu_{p}C_{ox}{\left(\frac{W}{L}\right)}_{2}\left(V_{DD}-V_{PI}-\left|V_{TH}\right|\right)$$

The current *I_d_*_2_ of the right half of the circuit can be obtained by Equation (30).

(30) $$I_{d2}=\mu_{p}C_{ox}{\left(\frac{W}{L}\right)}_{2}\left(V_{DD}-V_{PI}-\left|V_{TH}\right|\right)\cdot V_{drive-}$$

Due to the negative feedback inside the nonlinear multiplier module and the negative feedback in the driving loop, the current on both sides tends to *I_d_*_1_ = *I_d_*_2_, and the output driving feedback voltage of the nonlinear multiplier *V_drive−_* is obtained.

(31) $$V_{drive-}=\frac{V_{DD}-V_{CM}-\left|V_{TH}\right|}{V_{DD}-V_{PI}-\left|V_{TH}\right|}\cdot V_{dsen+}=A_{mul}V_{dsen+}$$

where *A_mul_* is the gain of the nonlinear multiplier. When the driving circuit starts quickly, and the driving displacement amplitude increases to the reference voltage *V_REF_*, the PI controller output voltage *V_PI_* < *V_CM_*. The nonlinear multiplier gain *A_mul_* < 1, and the nonlinear multiplier reduces the driving feedback voltage. As a result, the electrostatic driving force is reduced and the amplitude of driving displacement signal is reduced. If the driving displacement amplitude is too small, the PI controller output voltage *V_PI_* > *V_CM_*. When *A_mul_* > 1, the nonlinear multiplier will increase the driving feedback voltage. By increasing the electrostatic driving force, the amplitude of driving displacement signal is increased. The closed-loop negative feedback in the driving circuit can eliminate the static error between the amplitude of the driving detection signal and the reference voltage *V_REF_* of PI controller, so that the output of the PI controller (*V_PI_*) is equal to *V_CM_*. In this case, the gain of the nonlinear multiplier *A_mul_* = 1, and thus, Equation (32) can be obtained.

(32) $$V_{drive-}=V_{dsen+}$$

That is, the loop gain of the driving circuit is 1. The driving feedback voltage *V_drive−_* is in the same frequency and phase as the driving detection signal *V_dsen+_*, which satisfies the stability condition of the driving displacement amplitude of the gyroscope driving circuit.

### 4.3. Design of On-Chip Integrated Temperature Sensor

**Figure 14:**
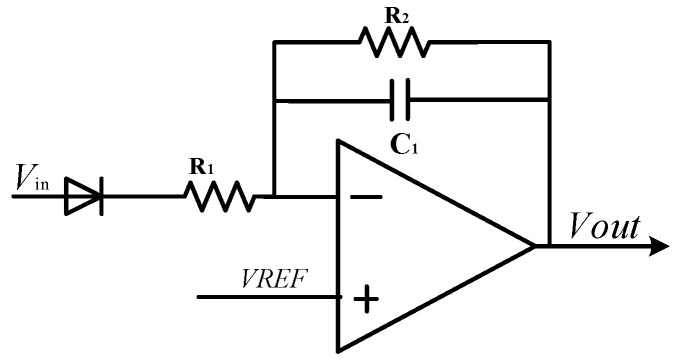
Positive temperature coefficient temperature sensor.

The change in ambient temperature will lead to the change in mechanical parameters of the gyroscope’s sensitive structural materials, which will lead to the change in gyroscope driving displacement amplitude. The change in sense mode sensitivity will be directly reflected in the output signal due to the change in the displacement amplitude driven by the gyroscope. This temperature drift will lead to errors between the angular velocity information detected by the interface circuit and the actual angular velocity input signal. Therefore, this design designs an on-chip integrated temperature sensor in the MEMS gyroscope interface circuit, and the integrated positive temperature coefficient temperature sensor is shown in Figure 14. When the ambient temperature is high, the sensitivity of the gyroscope’s sensitive structure decreases, resulting in the increase in the driving feedback voltage signal, which is represented by positive temperature coefficient. ADC voltage signal with positive temperature coefficient can be obtained by half-wave rectification and filtering using the gyroscope driving voltage signal for gyroscope temperature compensation.

**Figure 15:**
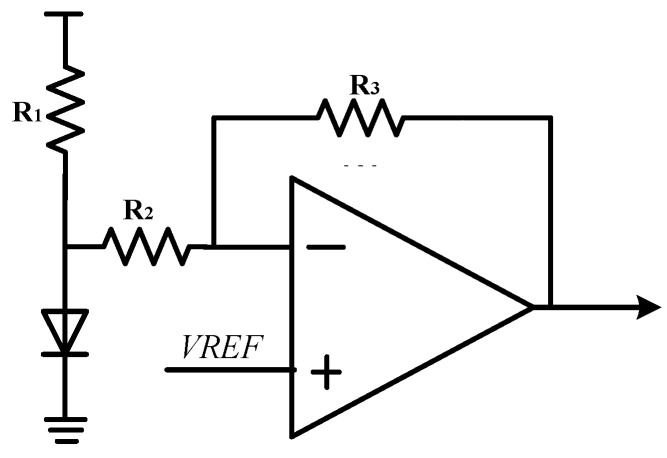
Negative temperature coefficient temperature sensor.

A negative temperature coefficient temperature sensor is integrated into the circuit based on the temperature characteristics of the diode on-voltage. Figure 15 shows the circuit structure of the temperature sensor. Similarly to the principle of band-gap reference circuit, the negative temperature coefficient of diode on-voltage can be used to compensate the high-order temperature change in the MEMS gyroscope. In addition, the inverter amplifying circuit can enhance the driving load capacity of the output signal of the temperature sensor.

**Figure 16:**
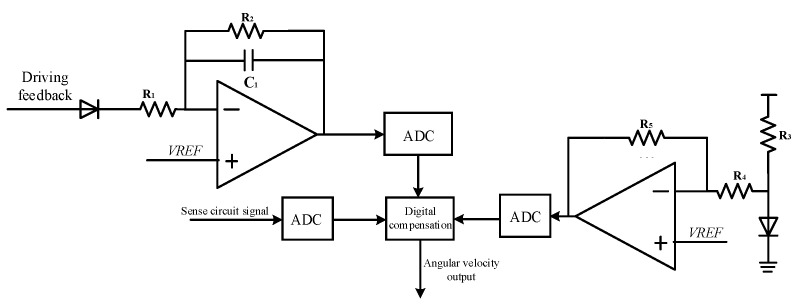
Circuit diagram of digital temperature compensation circuit module.

The positive and negative temperature coefficient signals generated by the temperature sensor will be converted into 1-bit stream signals after ΣΔ modulator, and the cascaded digital filter composed of CIC and IIR filter is converted into 12-bit digital signals. The digital temperature compensation circuit can be used to accurately compensate the change in output angular velocity caused by temperature offset in gyroscope sense circuit, so as to improve the detecting accuracy of the gyroscope interface circuit. Figure 16 shows the schematic diagram of the temperature compensation circuit system on the MEMS gyroscope sheet.

### 4.4. Circuit Design of ΣΔ Modulator

**Figure 17:**
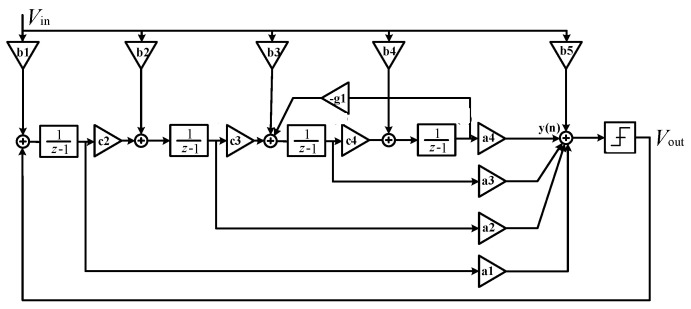
Schematic diagram of a fourth-order CIFF modulator.

ADC can convert the analog signal of the gyroscope output into the digital signal, and the modulator is one of the important circuit modules of ΣΔ ADC. In addition, the angular velocity signal after demodulation is converted to a digital signal, which is convenient for the post circuit to compensate the zero bias error and temperature drift of the gyroscope. ΣΔ ADC has the advantage of high accuracy compared to SAR ADC. Therefore, in this paper, fourth-order ΣΔ ADC diagonal velocity is used for digitization. In order to meet the requirements of this modulation design, a fourth-order CIFF structure is adopted in the design, and its ideal modeling is shown in Figure 17. When the selected oversampling rate OSR is 128, the maximum SNR theoretically achieved by the modulator is 121.3 dB.

**Figure 18:**
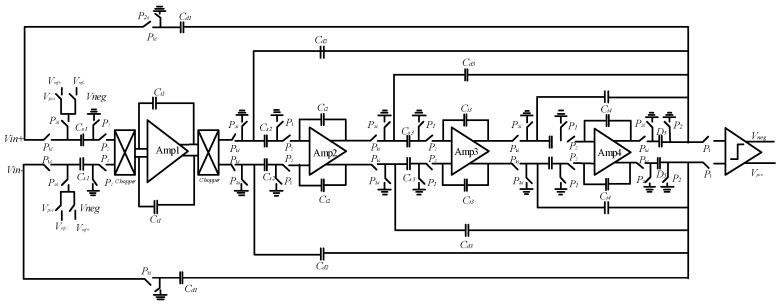
Circuit diagram of a fourth-order CIFF ΣΔ modulator.

The modulator is designed with a fourth-order 1-bit quantized ΣΔ CIFF structure. The circuit structure of the modulator is shown in Figure 18. The full difference structure is used to suppress even harmonic distortion. Due to the *KT*/*C* noise of the first stage capacitor of the modulator, the circuit integration and sampling capacitance are optimized to reduce the area of the modulator layout. In order to reduce the power consumption, a new working mode of the circuit system is proposed. The sampling capacitance and feedback signal sampling capacitance are separated to greatly reduce the harmonic distortion of the system. The first stage integrator increment chopper circuit can modulate 1/*f* noise outside the signal bandwidth, thereby improving the SNR in the low-frequency range of the ΣΔ modulator.

**Figure 19:**
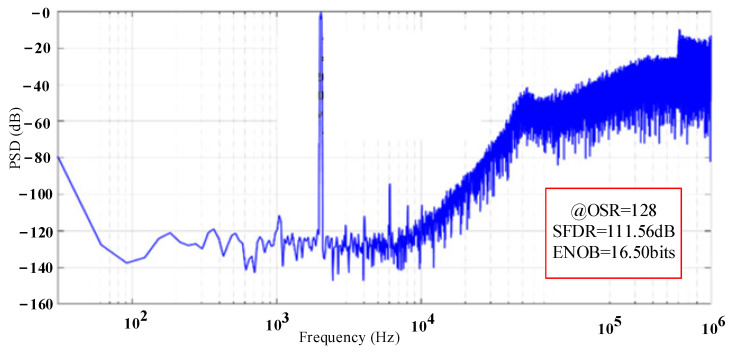
Power spectral density of the ΣΔ modulator.

By inputting the AC signal source to the modulator circuit, the modulator is simulated at the transistor level. The input power supply voltage of the modulator is 5 V, and the clock frequency is 2 MHz. After simulation, the number of sampling points is 65,536, and the chop frequency of the op-amp is 500 kHz. The signal bandwidth is 7.8 kHz, and the input signal amplitude is 2 V. The frequency is 2.01 kHz sinusoidal signal. Figure 19 shows the output power spectral density of the modulator. The ΣΔ modulator noise floor is about −135 dB/√(Hz), signal-to-noise distortion ratio is −111.56 dB, and the performance of the modulator meets the design requirements.

## 5. Experimental Results and Discussion of Gyroscope

**Figure 20:**
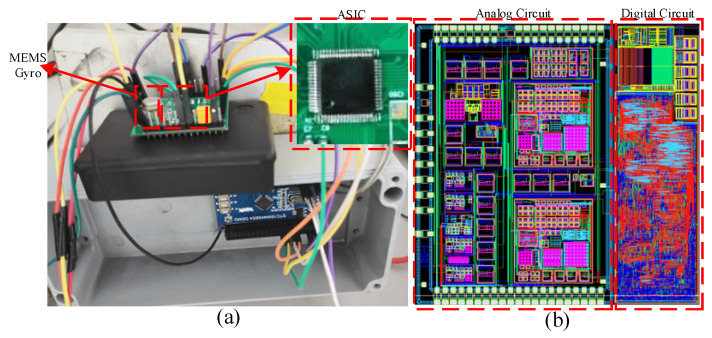
MEMS gyroscope test circuit platform diagram: (**a**) MEMS gyroscope test circuit; (**b**) MEMS gyroscope ASIC layout.

In order to verify the performance of the MEMS gyroscope interface ASIC, an interface ASIC of the MEMS gyroscope interface circuit is designed by 0.18 um standard CMOS BCD process. In order to test the performance of the MEMS gyroscope system, the MEMS gyroscope system is tested and analyzed at a temperature of 25 °C and a power supply voltage of 5 V. Gyroscope interface ASIC area is 5.7 mm^2^ (2.4 × 2.4 mm), and its power consumption is 25 mW. The test circuit diagram of the MEMS gyroscope is shown in Figure 20.

**Figure 21:**
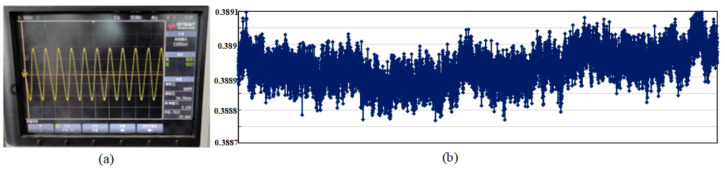
MEMS gyroscope driving stability measurement results: (**a**) Driving detection voltage signal; (**b**) Driving test signal stability measured result.

Figure 21 shows the test diagram of driving voltage signal of **the** MEMS gyroscope. It can be found that after the ASIC is powered on, the whole MEMS gyroscope system can vibrate quickly and achieve stable simple harmonic vibration, and the resonant frequency of the gyroscope driving circuit is 54.76 kHz. Both the driving detection signal and the driving feedback signal oscillate in constant amplitude at the gyroscope resonant frequency. At the room temperature of 25 °C, use a multimeter to measure the AC amplitude of the driving voltage signal at five points per second. The result after one hour is collected, as shown in Figure 21. After calculation, the average RMS value of driving detection signal is 0.387 V. The maximum value of driving voltage signal amplitude is 0.389 V and the minimum value is 0.389 V. By averaging every 50 points of the obtained test data, the variance of driving the amplitude fluctuation of the detection signal can be calculated as 2.47 × 10^−5^ V. The results show that the driving displacement amplitude of the MEMS gyroscope has good stability.

**Figure 22:**
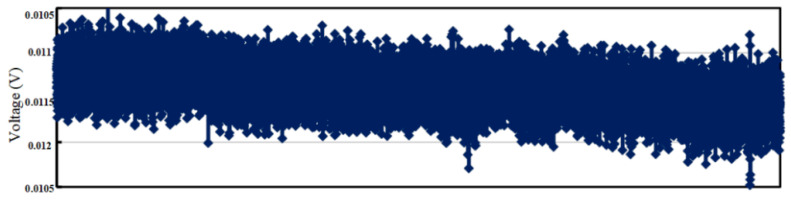
Test result of MEMS gyroscope zero bias output.

Figure 22 shows the stability test result of the output angular velocity signal of the MEMS gyroscope detecting circuit. It can be obtained that a bias instability of the MEMS gyroscope is 5.1°/h by standard Allen variance.

**Figure 23:**
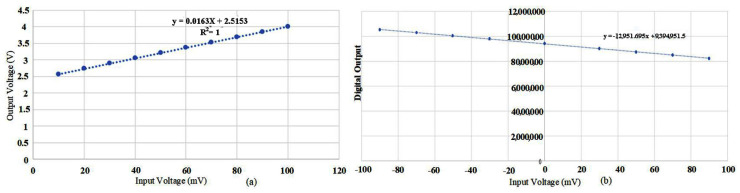
MEMS gyroscope test circuit input and output test results: (**a**) The analog input and output result; (**b**) The digital input and output result.

Scale factor is the most important performance parameter of the MEMS gyroscope, reflecting the linearity of the gyroscope output. In order to simulate the actual angular velocity input of the actual gyroscope, an AC signal with the same frequency as the resonant frequency is applied to the sense circuit of the MEMS gyroscope interface circuit. Figure 23 shows the linear fitting curve between the output signal and input voltage of the MEMS gyroscope test circuit. It can be obtained that the nonlinearity of the output angular velocity detected by the MEMS gyroscope is 0.03%.

Table 2 compares this work with MEMS gyroscopes with different interface circuit architectures and corresponding functions. The whole functional module of the ASIC designed in this paper, including the gyro interface circuit, is realized by analog and digital hybrid circuit integration. In addition, the on-chip temperature compensation method is used to realize the zero bias correction of the gyroscope. The multistage cascade digital filter is adopted to avoid the redundancy and waste of circuit components and improve the availability of device effectively. It can be seen from Table 2 that the interface ASIC of the MEMS gyroscope designed in this work has satisfactory system performance, especially in terms of output nonlinearity.

## 6. Conclusions

This paper presents a design scheme of interface ASIC for digital output of a MEMS vibrating gyroscope. The system uses an analog closed-loop self-excited drive circuit with AGC to realize rapid oscillation and precise drive of the gyro. Compared with the open-loop drive circuit scheme, the closed-loop driving scheme can obtain higher drive stability. In this paper, the electrical equivalent model of the sensitive structure of the MEMS vibrating gyroscope is established, and the simulation results of the system-level model are further discussed, and the feasibility of the gyroscope closed-loop drive and digital output design scheme is verified. This work will contribute to the research, system-level modeling and circuit implementation details of the MEMS vibrating gyro integrated circuits. The on-chip integration of analog front end and digital temperature compensation circuit is realized, the area of interface circuit is reduced, and the power consumption of the gyro system is reduced in this work. The interface ASIC is manufactured using a standard 0.18 μM CMOS BCD process. The experimental results show that the full range of the system output of the gyroscope is ±200°/s, the system bandwidth is 100 Hz, the bias instability is 5.1°/h and the nonlinearity is 0.03%.

## Figures and Tables

**Table 1:** Correspondence between gyroscope physical parameters and equivalent electrical parameters.

Sensitive to Structural Physical Parameters	Equivalent Electrical Parameters
Velocity *v*	Current *i*
Displacement *x* in the driving direction	The quantity of charge *Q_d_* on capacitor *C*
Effective mass *m*	Inductance *L*
Damping coefficient *b*	Resistance *R*
Elastic coefficient *k*	Capacitance *C*
Feedback driving force *F_d_* /Coriolis force *F_c_*	Constant pressure source *U*
Resultant force	Voltage droop on the inductor *U_L_*
Damping force *F_b_*	Voltage droop on the resistor *U_R_*
Elastic force *F_k_*	Voltage droop on the capacitor *U_C_*

**Table 2:** Performance comparison and summary.

**Properties**	[32]	[33]	[34]	[35]	This Work
Year	2014	2016	2017	2020	2022
Circuit type	PCB + FPGA	Analog + FPGA	ASIC	PCB	ASIC
Power (mW)	-	27.3	21	-	25
Chip area (mm^2^)	-	2.3	7.3	-	5.7
Input range (°/s)	-	±100	500	±200	±200
Bandwidth (Hz)	-	80	480	-	100
Bias instability (°/h)	1.5	0.5 V	5.5	11.3	5.1
Nonlinearity (%)	0.15	0.1	-	-	0.03

## Data Availability

Not applicable.
